# The adaptive market hypothesis and high frequency trading

**DOI:** 10.1371/journal.pone.0260724

**Published:** 2021-12-17

**Authors:** Ke Meng, Shouhao Li

**Affiliations:** School of Public Policy and Management, Tsinghua University, Beijing, China; The Bucharest University of Economic Studies, ROMANIA

## Abstract

This paper uses NASDAQ order book data for the S&P 500 exchange traded fund (SPY) to examine the relationship between one-minute, informational market efficiency and high frequency trading (HFT). We find that the level of efficiency varies widely over time and appears to cluster. Periods of high efficiency are followed by periods of low efficiency and vice versa. Further, we find that HFT activity is higher during periods of low efficiency. This supports the argument that HFTs seek profits and risk reduction by actively processing information, through limit order additions and cancellations, during periods of lower efficiency and revert to more passive market-making and rebate-generation during periods of higher efficiency. These findings support the argument that the adaptive market hypothesis (AMH) is an appropriate description of how prices evolve to incorporate information.

## Introduction

In this paper, we test the efficient market hypothesis (EMH) using NASDAQ ITCH-feed data on the S&P 500 exchange traded fund (SPY). We find that support for the EMH’s weak form, where price changes fully incorporate past price changes, varies widely over one-minute timeframes. Efficiency appears to cluster. There are periods of very low and very high weak-form efficiency that alternate back-and-forth. In our review, this paper is the first to document evidence supporting the evolving nature of efficiency at this resolution. Consistent with the existing studies [1–5], our findings suggest that the adaptive market hypothesis (AMH) is a better description of intra-day SPY returns.

Because trading activity at one-minute resolution is driven by high frequency traders (HFTs), we also study whether their limit order additions and cancelations are related to the level of market efficiency. We find that there is a negative correlation between the two. When markets are highly efficient, HFTs tend to be less active. When markets are less efficient, HFTs tend to be more active. While the variability explained is small, this relationship is very statistically significant. This supports the argument that HFTs seek to profit and reduce risk by removing short-term inefficiencies [6]. Thus, HFTs appear to adapt quickly to the evolving level of market efficiency as a means of survival, as suggested by some scholars [7, 8]. In our review, this paper is the first to document the relationship between the level of support for the EMH and the level of HFT activity.

This paper makes three contributions. First, this paper examines the predictability of returns using one-minute time series data extracted from message level data on the SPY. Following Urquhart and McGroarty [5], we use several tests of predictability as proxies for tests of the EMH using both our entire data set (i.e. either/or) and overlapping sub-sample windows (i.e. time varying continuum). These reveal the time-varying level of efficiency. Second, we apply Blocher *et al*.’s measure of HFT activity to the limit order data and examine the relationship between HFT activity and the level of support for each of the tests [9]. Third, we develop a novel methodology to measure minute-by-minute limit order activity to associate high and low spikes in HFT activity with the outcomes of the tests.

The remainder of this paper is organized as follows. The second section provides background and a literature review. The third section describes the data, statistical measures, and methodology. The fourth section presents the results and discussion. The fifth and final section provides concluding remarks.

## Background and literature review

Since Fama [10, 11] first proposed the EMH, a large body of research has challenged its three forms. Theoretically, Grossman and Stiglitz argue that the EMH cannot hold because we can observe that market participants invest in information, something they would not do if it was not profitable [12]. Empirically, many authors find evidence the weak form of the EMH does not hold. Fama, himself, finds evidence of such inefficiency [13, 14]. Jegadeesh and Titman, and Carhart show an anomaly that momentum is a predictor of future returns [15, 16]. Lo and MacKinlay tests the EMH using statistical methods, and they strongly reject the random walk hypothesis [17]. However, as the EMH is a categorical theory, methods employed in these studies naturally seek an “either/or” result over a historical set of data [18].

Lo attempts to reconcile the EMH with the empirical evidence by proposing a continuum theory, the AMH [19, 20]. Using a biological analogy, the AMH suggests that the market consists of an evolving set of trading strategies, so that the aggregate level of efficiency also continuously evolves. From this perspective, Lim and Brooks suggest that empirical support for the EMH is time-varying and “can be rationalized within the framework of the AMH” [21]. A growing literature supports this view [5, 22–30]. Importantly for this paper, Alvarez-Ramirez, *et al*. finds evidence of time-varying efficiency and that prices are more efficient over shorter horizons [31]. This supports findings of earlier studies, such as Battalio, *et al*., Chordia, *et al*., and Zhao and Chung, which find that order execution speed is an important component of market efficiency [32–34]. Chalamandaris reports that traders adapt to macroeconomic events and thus vary the level of market efficiency [35].

Today, HFT dominates trading volume, accounting for roughly 70% of activity [36–38]. A growing body of research investigates the impact of HFT on liquidity, bid-ask spreads, volatility, and efficiency [39–42]. They basically find that HFT activity is associated with narrower spreads, lower volatility, and greater liquidity. Blocher, *et al*. finds that HFT activity leads to executions, largely driven by liquidity demanding lower frequency traders, that do not occur at informationally incorrect (i.e. inefficient) prices [9].

From our review, however, there are few empirical connections in the literature between the activity of HFTs and the AMH. We suspect that accessing message level market data is the primary obstacle. Those that do make the connection include: Virgilio, which finds that HFT allows a few fast traders to profit from arbitrage and thus falsifies the EMH [43]; Manahov and Hudson, which uses simulation to demonstrate that a larger market with more heterogenous traders is the key to increased efficiency [3]; and Manahov, *et al*., which concludes that heuristics enable artificial traders to adapt to changing market environments [7]. Recently, research on the AMH in nascent cryptocurrency markets has become popular. Chu *et al*. find that activity in these markets support the AMH [44]. These papers—Chu, *et al*. and Manahov, *et al*.—agree that studying HFT and the AMH properly requires greater granularity in the data, something not always readily available [7, 44]. We benefit from having access to message level exchange data, and this enables us to make an empirical contribution that connects HFT to the AMH on a meaningful timescale.

## Data, statistical measures, and methodology

Our data set consists of the NASDAQ ITCH-feed data for the S&P 500 Index exchange traded fund (SPY) from January 1 to December 31, 2012. The 48 gigabytes of raw data contain every message about additions to and cancelations from the limit order book as well as executions, time-stamped to the nanosecond. We use this raw data to measure the level of HFT activity. For the purposes of testing price predictability, we first eliminate microstructure effects by calculating the bid-ask mid-point price (i.e. (bid price + ask price)/2) at each one-minute interval in the raw data and then extract the time series of one-minute log mid-point price changes. This is a timescale at which HFTs are active. Using the mid-point price avoids issues associated with bid-ask bounce [36]. Our time series of price changes consisted initially of roughly 98,280 data points (390 minutes per trading day × 252 trading days), but after cleaning and adjusting for partial trading days, the final data set consisted of 97,110 data points.

From the raw data, we calculate two measures of HFT activity, based on its penchant for adding and cancelling limit orders [41, 45–48]. The first measure follows Blocher, *et al*. to identify cancelations [9]. We define an indicator variable *I*, where *I*_*b*_ = 1 if the event is a cancel of a bid, and *I*_*b*_ = 0 if it is not. Likewise, for *I*_*a*_ on the ask side. We then calculate an exponentially weighted moving average (EWMA) of the transformed event series. For the *i*th observed message on the bid side:

$${b\_inf}_{i}={b\_inf}_{i-1}+\frac{2}{11}\times (I_{b}-{b\_inf}_{i-1})$$ (1)

and for the ask side:

$${a\_inf}_{i}={a\_inf}_{i-1}+\frac{2}{11}\times (I_{a}-{a\_inf}_{i-1})$$ (2)

where 2 / 11 is the EWMA equivalent of a simple moving average of 10 events.

After the above modification, we average the EWMA for each one-minute interval:

$${mb\_inf}_{t}=\frac{1}{{Nb}_{t}}\times \sum_{i=1}^{{Nb}_{t}}{b\_inf}_{i}$$ (3)

$${ma\_inf}_{t}=\frac{1}{{Na}_{t}}\times \sum_{i=1}^{{Na}_{t}}{a\_inf}_{i}$$ (4)

where *t* = 1 to *T*, the size of sample. *Nb*_*t*_ and *Na*_*t*_ are the total number of limit orders on the bid and ask sides respectively at time *t*. We then average these two equations as:

$${m\_inf}_{t}=\frac{1}{2}\times ({mb\_inf}_{t}+{ma\_inf}_{t})$$ (5)

so that *m_inf*_*t*_ is the measure of the level of HFT activity for each one-minute interval.

The second measure of HFT activity, newly defined in this paper, finds high and low spikes in HFT activity as measured by *m_inf*_*t*_ in (5). For every 30-minute interval over 250 trading days, we capture HFT activity minute-by-minute. This results in 3,237 half-hour intervals each containing 30 one-minute periods. Within each half hour, we count the number of one-minute outlier observations of HFT activity, measured as those that violate the 1.5 interquartile spread threshold greater or lower than the median of HFT activity (interquartile spread, aka IQS, is calculated as the difference between the upper and lower quartile, for each half hour. It is pragmatic to use quartile since the data is skewed.). That is, we normalize by the specific IQS for each half-hour interval (say 9:00 to 9:30 am) across all 250 trading days. We call these violations of the IQS high and low “spikes.” The hypothesis is that there will be more positive outliers (i.e. high spike counts) of HFT activity for periods with lower support for market efficiency, and conversely so for negative outliers (i.e. low spike counts). Table 1 contains descriptive statistics for the time series of mid-point log returns. While the Mean *Return* over the one-minute interval is nearly zero, 0.0001%, there is StdDev, Skewness, and Kurtosis.

**Table 1:** Descriptive statistics.

Variable	N	Mean	Std Dev	Minimum	Maximum	Skewness	Kurtosis
*Return*	97109	0.0001	0.0416	-2.76712	1.72846	-4.60614	477.830
*m_inf*	97110	0.4714	0.0243	0.09467	0.77722	-0.86364	15.9428
*ma_inf*	97110	0.4713	0.0298	0.01225	0.93046	-0.13012	17.6076
*mb_inf*	97110	0.4714	0.0302	0.00064	0.85291	-0.80685	20.7161
*Spread*	97110	0.0000	0.0000	0.00007	0.00085	11.9757	231.922
*Liquidity*	97110	0.6002	0.0396	0.28247	0.79184	-0.29815	1.17072
*Trading Volume*	97110	15.801	1.2884	0.00000	19.49643	-5.49500	62.6300
*VIX*	97110	3.42858	0.44302	2.70538	4.29676	0.04786	-1.25991

We use the time series data in two ways to test the EMH. First, we use the entire time series of returns. Second, we create sub-samples of overlapping windows of 500 returns, which follows Kim, *et al*. [1]. The tests we perform on each of these two datasets are Lo and MacKinlay’s variance ratio test (VRT) [17], the Chow and Denning (1993) test (CDT) [49], and the Wright (2000) joint rank (WJR) and joint sign (WJS) test with bootstrapping [50]. The VRT addresses the random walk hypothesis over the whole sequence of returns [51] (see Charles and Darne, 2009). However, to assess any variation in market efficiency over time, the VRT is insufficient. We use the CDT, which allows for multiple periods [51] (see Charles and Darne, 2009). The WJR and WJS are non-parametric and thus have higher power to overcome serial correlation, and WJR assumes i.i.d while WJS assumes both i.i.d and martingale difference [52]. All these tests are linear, and we have tried to address any non-linearity in the returns using Brock *et al*.’s test (BDST) [53], which has been applied to lower frequency trading. The BDST is a non-parametric test for non-linear, serial dependence that requires first whitening the data. However, our attempts to run the BDST on our higher frequency data were unsuccessful. Despite testing up to 20 lags of the auto-regressive model, the random error terms could not pass the Ljung-Box test up to 10 lags. Thus, we have left this test out. In general, using multiple tests with differing logics ought to help reach a robust conclusion [5, 54]. The following subsections present details of the various tests.

### Variance Ratio Test (VRT)

The widely used variance ratio test of Lo and MacKinlay is straightforward [17]. If a market is weak-form efficient, then the return series *r*_*t*_ where *t* = {0, 1, 2, …, *T*} will not be serially correlated. If this is the case, then the variance of some *k*-period return *σ*_*k*_^2^ will be the same as *k* times the variance of one period return *σ*^2^.

Thus, a ratio of the two variances should equal one as:

$$VR(k)=\frac{\sigma_{k}^{2}}{k\sigma^{2}}$$ (6)

where *VR*(*k*) follows an *F*-distribution. If the outcome of the test is significantly different from one, then the hypothesis that the returns follow a random walk is rejected. We can rewrite (6) as:

$$VR(k)=1+2\sum_{j=1}^{k-1}\left(1-\frac{j}{k})\rho (j\right)$$ (7)

where *ρ*(*j*) is the order *j* autocorrelation coefficient of *r*_*t*_. If *VR*(*k*) = 1, then *ρ*(*j*) = 0 for all *j*’s. If the value of *VR*(*k*) is greater (less) than one, then there is positive (negative) serial correlation.

For robustness we use both of Lo and MacKinley’s proposed statistics—*M*_1_(*k*), which assumes *r*_*t*_ is i.i.d., and *M*_2_(*k*), which assumes *r*_*t*_ is heteroscedastic. In both cases, these are asymptotically standard normally distributed as *T* increases. These are defined as:

$$M_{1}(k)=\frac{VR(k)-1}{\sqrt{\omega (k)}}$$ (8)

where the asymptotic variance *ω*(*k*) is determined as:

$$\omega (k)=\frac{2(2k-1)(k-1)}{3kT}$$ (9)

and:

$$M_{2}(k)=\frac{VR(k)-1}{\sqrt{\theta (k)}}$$ (10)

where the asymptotic variance *θ*(*k*) is given as:

$$\theta (k)=\sum_{j=1}^{k-1}{\left[\frac{2(k-j)}{k}\right]}^{2}\varphi (j)$$ (11)

$$\varphi (j)=\frac{\left\{\sum_{t=j+1}^{T}{\left(r_{t}-\hat{\mu }\right)}^{2}{\left(r_{t-j}-\hat{\mu }\right)}^{2}\right\}}{{\left[\sum_{t=1}^{T}{\left(r_{t}-\hat{\mu }\right)}^{2}\right]}^{2}}$$ (12)

$$\hat{\mu }=\frac{1}{T}\sum_{t=1}^{T}r_{t}$$

Following the existing studies, we evaluate *k* = {2, 4, 8, 16, 32, 64} for testing the EMH with the whole sample [5, 55]. Typically, *k* = {2, 5, 10, 30} for daily data or *k* = {2, 4, 8, 16} for weekly data [55]. Since we are the first to test the EMH for HFT, for robustness we include more values of *k*. If one of the *k*’s gets rejected, we reject the random walk hypothesis. From *M*_1_(*k*) and *M*_2_(*k*), which are our *z*-values, we find the corresponding *p*-values under the standard normal.

### Chow-Denning Test (CDT)

Chow and Denning argue that VRT results reject the EMH too easily using a set of *k*’s with one or two significant results [49]. They expand the VRT into an omnibus test, which takes only the *M*_1_(*k*) and *M*_2_(*k*) with the maximum absolute values as:

$${CD}_{1}=\sqrt{T}\;\underset{1\leq j\leq m}{\max}\;\left|M_{1}(k_{j})\right|$$ (13)

and:

$${CD}_{2}=\sqrt{T}\;\underset{1\leq j\leq m}{\max}\;\left|M_{2}(k_{j})\right|$$ (14)

Both statistics *CD*_1_ and *CD*_2_ follow a studentized maximum modulus distribution with *m* and *T* degrees of freedom. Their significance can be found in statistical tables for this distribution and we evaluate the null hypothesis of a random accordingly. The Chow-Denning Test is thus a joint hypothesis test that all the variance samples within a specified range scale linearly.

### Wright Joint Rank and Sign Tests (WJR and WJS)

The VRT and the CDT are parametric and based on asymptotic variance, which implies that they do not perform well given finite samples. For this reason, Wright suggests two generic tests, one using the rank, and the other the sign of the return series rather than the values [50]. This could be more powerful for models with more features, such as serial correlation and fractionally integrated alternatives [52]. We set the ranks of log returns *r*_*t*_ as *r*(*r*_*t*_). If *r*_*t*_ is i.i.d., then the ranks are random permutations of the numbers *t* = 1, 2, …, *T*, each with equal probability. We standardized the ranks as:

$$r_{1,t}=\frac{r(r_{t})-\frac{T+1}{2}}{\sqrt{(T-1)(T+1)/12}}$$ (15)

and:

$$r_{2,t}=\Phi^{-1}[\frac{r(r_{t})}{T+1}]$$ (16)

where *Φ*^-1^ is the inverse of the standard normal cumulative distribution function. The Wright rank test is defined as:

$$R_{1}(k)=(\frac{{\left(Tk\right)}^{-1}\sum_{t=k}^{T}{\left(r_{1,t}+\cdots +r_{1,t-k+1}\right)}^{2}}{T^{-1}\sum_{t=1}^{T}r_{1,t}^{2}}-1)\times {\omega (k)}^{-1/2}$$ (17)

$$R_{2}(k)=(\frac{{\left(Tk\right)}^{-1}\sum_{t=k}^{T}{\left(r_{2,t}+\cdots +r_{2,t-k+1}\right)}^{2}}{T^{-1}\sum_{t=1}^{T}r_{2,t}^{2}}-1)\times {\omega (k)}^{-1/2}$$ (18)

where *ω*(*k*) is in (9), and where *R*_1_ and *R*_2_ follow the same sampling distribution, and their critical values can be obtained through simulation. This generic test is still single period, and we select the holding period *k* for our tests. Although these tests only vary in whether one standardizes against the uniform or normal distribution we report both methods. Belaire-Franch and Contreras propose a similar version of CDT for the WJR test considering all holding periods selected as [52]:

$${JR}_{1}=\underset{1\leq i\leq m}{\max}\;\left|R_{1}(k_{i})\right|$$ (19)

$${JR}_{2}=\underset{1\leq i\leq m}{\max}\;\left|R_{2}(k_{i})\right|$$ (20)

For the Wright sign test, the setting is:

$$S(k)=(\frac{{\left(Tk\right)}^{-1}\sum_{t=k}^{T}{\left(s_{t}+\cdots +s_{t-k+1}\right)}^{2}}{T^{-1}\sum_{t=1}^{T}s_{t}^{2}}-1)\times {\omega (k)}^{-1/2}$$ (21)

where *s*_*t*_ = 2*u* (*r*_*t*_, 0) and *u* (*r*_*t*_, 0) = 0.5 if *r*_*t*_ > 0 or -0.5 otherwise, and the return series is heteroscedastic. There is another version of the joint sign test but is less powerful [1, 5, 50, 52, 54]. Thus we choose not to use it. As before, following Belaire-Franch and Contreras we define the joint sign test [52]:

$$JS=\underset{1\leq i\leq m}{\max}\;\left|S(k_{i})\right|$$ (22)

where the critical values *JS* can be obtained by simulation.

### Details of the market efficiency testing

We explore the AMH by dividing the data set into overlapping sub-samples since support for AMH requires results that show an evolution of efficiency over time. Our measures of market efficiency are the rolling *p*-values of these statistics defined in Section 3.2. To calculate these for the CDT, we use the wild bootstrap method proposed by Kim to pin down the finite sample bias [56]. We apply the wild bootstrap method in four steps:

**STEP 1:** We generate the bootstrap sample *r*_t_* = *r*_*t*_ *δ*_*t*_ (*t* = 1, 2, …, *T*) where *δ*_*t*_ is a sequence of random errors with zero mean and unit variance, using the standard normal distribution [56].
**STEP 2:** Using the bootstrap sample *r*_t_*, we calculate Kim and Shamsuddin’s test statistic MV* from Eqs (8)–(22) [57].
**STEP 3:** We repeat STEP 1 and STEP 2 *n* times to form a bootstrap distribution of the test statistic {MV*(*j*)}^*n*^_*j* = 1_.
**STEP 4:** We calculate the *MV* statistic using the original data. The bootstrap distribution {MV*(*j*)}^*n*^_*j* = 1_ is used as the sampling distribution of *MV*. The *p*-value of the bootstrap test is estimated as the proportion of {MV*(*j*)}^*n*^_*j* = 1_ larger than the *MV* statistic.

## Results and discussions

In this section we report the results using various holding periods *k*. For the entire sample, we report the VRT results, and for the rolling tests, we report the results for the CDT, WJR, and WJS.

### Results of the EMH test using the entire time series

Panel A of Table 2 shows the results of the VRT using *k* = {2, 4, 8, 16, 32, 64} for both i.i.d. and non-i.i.d. random error terms. As can be seen, all holding periods generate *p*-values at the < 0.0001 level. These results lead us to reject the random walk hypothesis. Panel B of Table 2 shows the results of the CDT. We include results for both homoscedastic (*CD*_1_) and heteroscedastic (*CD*_2_) assumptions. For robustness, we also included the larger holding period of *k* = 128 in addition to those in Panel A. Both the *CD*_1_ and *CD*_2_ *p*-value results are < 0.0001. This also leads us to reject the random walk hypothesis. Panel C of Table 2 shows the results of the WJR1, WJR2, and WJS tests. All three *p*-values are < 0.01. These results also lead us to reject the random walk hypothesis. However, simply rejecting the week-form EMH over a time period does not necessarily provide evidence in support of the AMH. By examining the rolling *p*-values of the overlapping windows from the tests, we can see how market efficiency evolves over time.

**Table 2:** The Results of the four tests using the entire data set.

**Panel A: Results of VRT**		
**Holding Period *k***	**Random Error**	***p*-value**
2	i.i.d	< 0.0001
4	i.i.d	< 0.0001
8	i.i.d	< 0.0001
16	i.i.d	< 0.0001
32	i.i.d	< 0.0001
64	i.i.d	< 0.0001
2	non-i.i.d	< 0.0001
4	non-i.i.d	< 0.0001
8	non-i.i.d	< 0.0001
16	non-i.i.d	< 0.0001
32	non-i.i.d	< 0.0001
64	non-i.i.d	< 0.0001
**Panel B: Results of CDT**		
**Random error setting**	**Statistics**	***p*-value**
CDT (*CD*_1_)	87.3798	< 0.0001
CDT (*CD*_2_)	8.073	< 0.0001
**Panel C: Results of WJR and WJS**		
**Tests**	**Statistics**	***p*-value**
WJR (*JR*_1_)	12.3	< 0.01
WJR (*JR*_2_)	9.84	< 0.01
WJS (*JS*)	42.6	< 0.01

### Results of the EMH test using the overlapping windows

**Fig 1:**
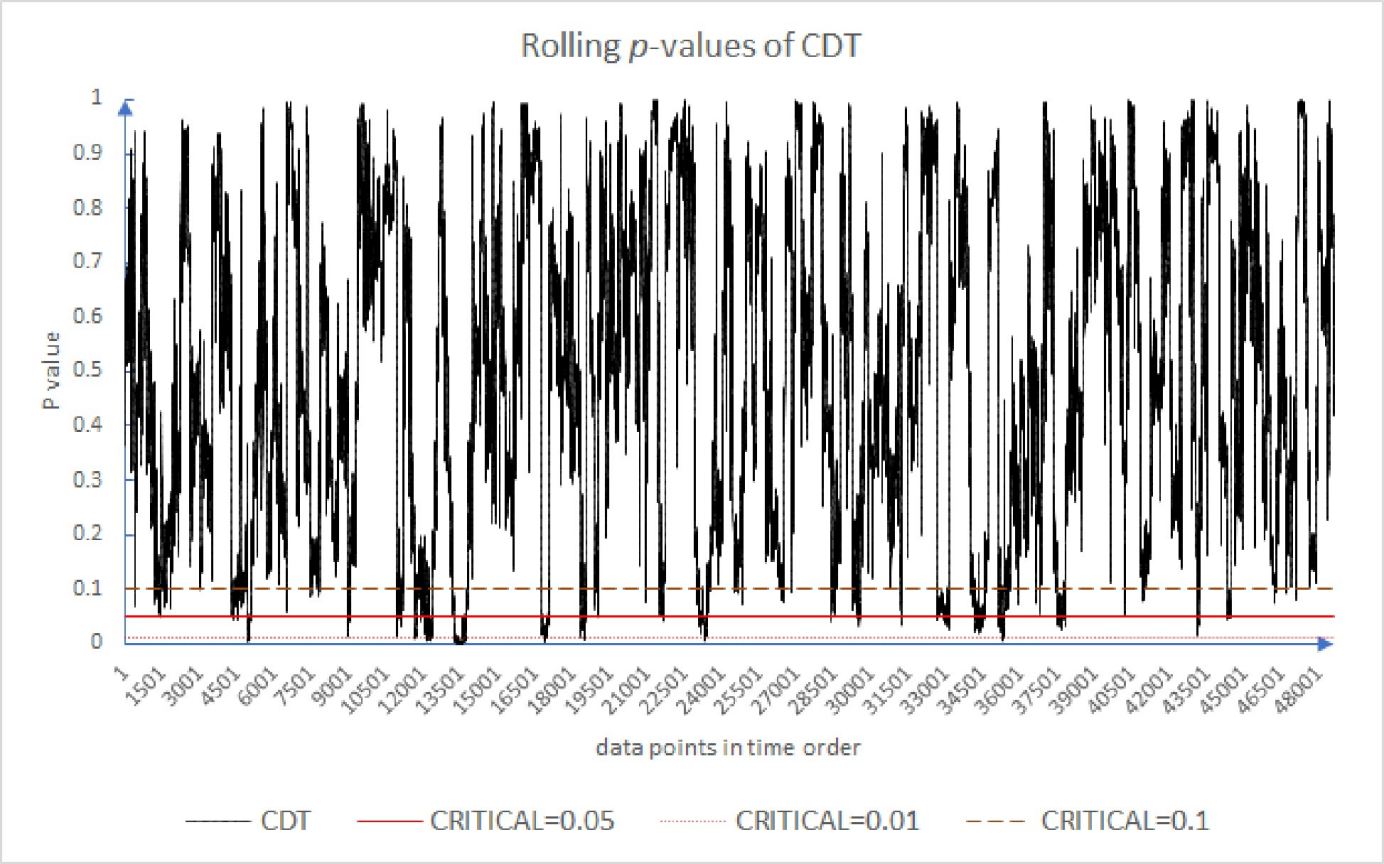
Rolling *p*-values of the CDT test for the first half of 2012.

**Fig 2:**
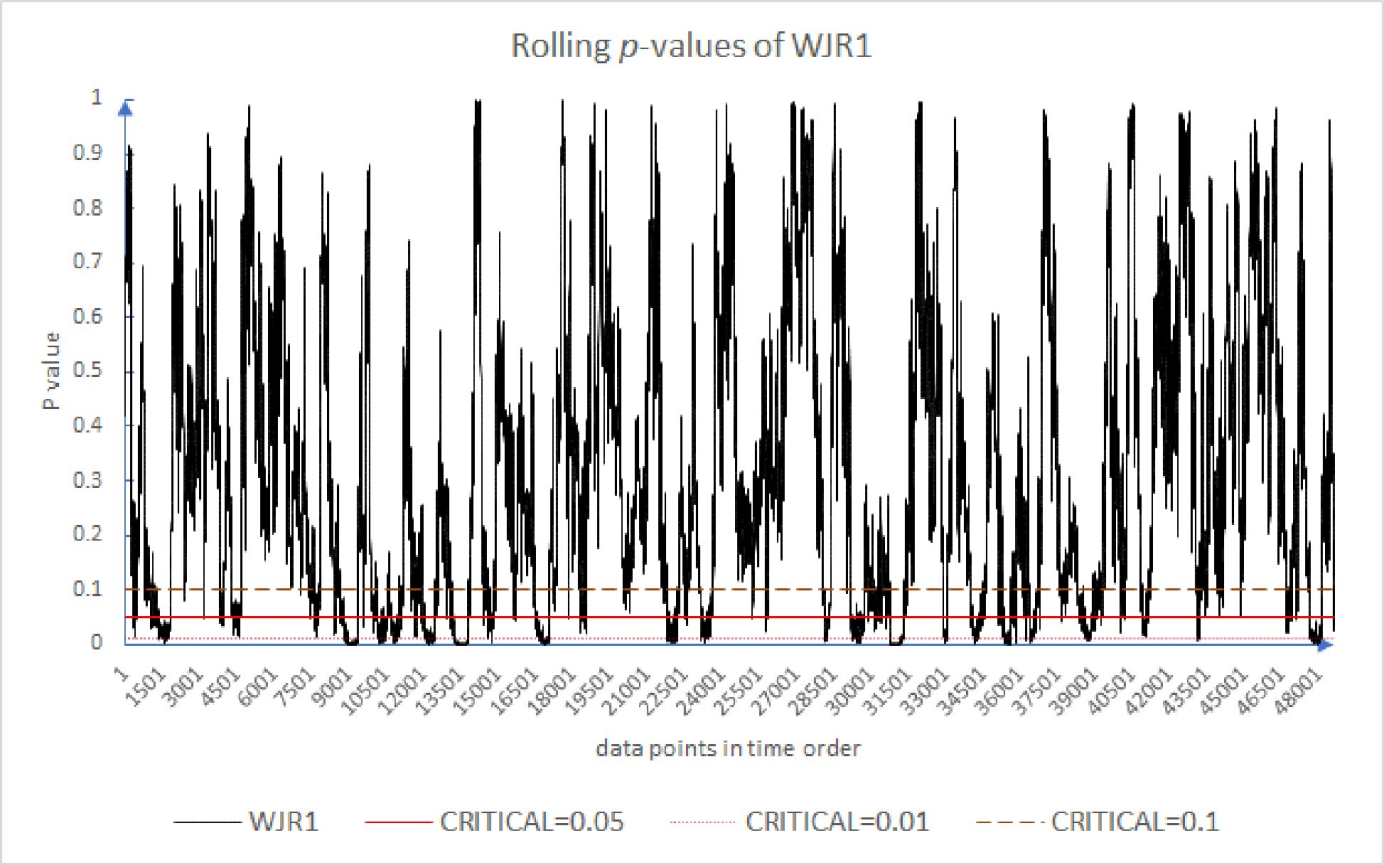
Moving *p*-value of WJR1 test for the first half year of 2012.

**Fig 3:**
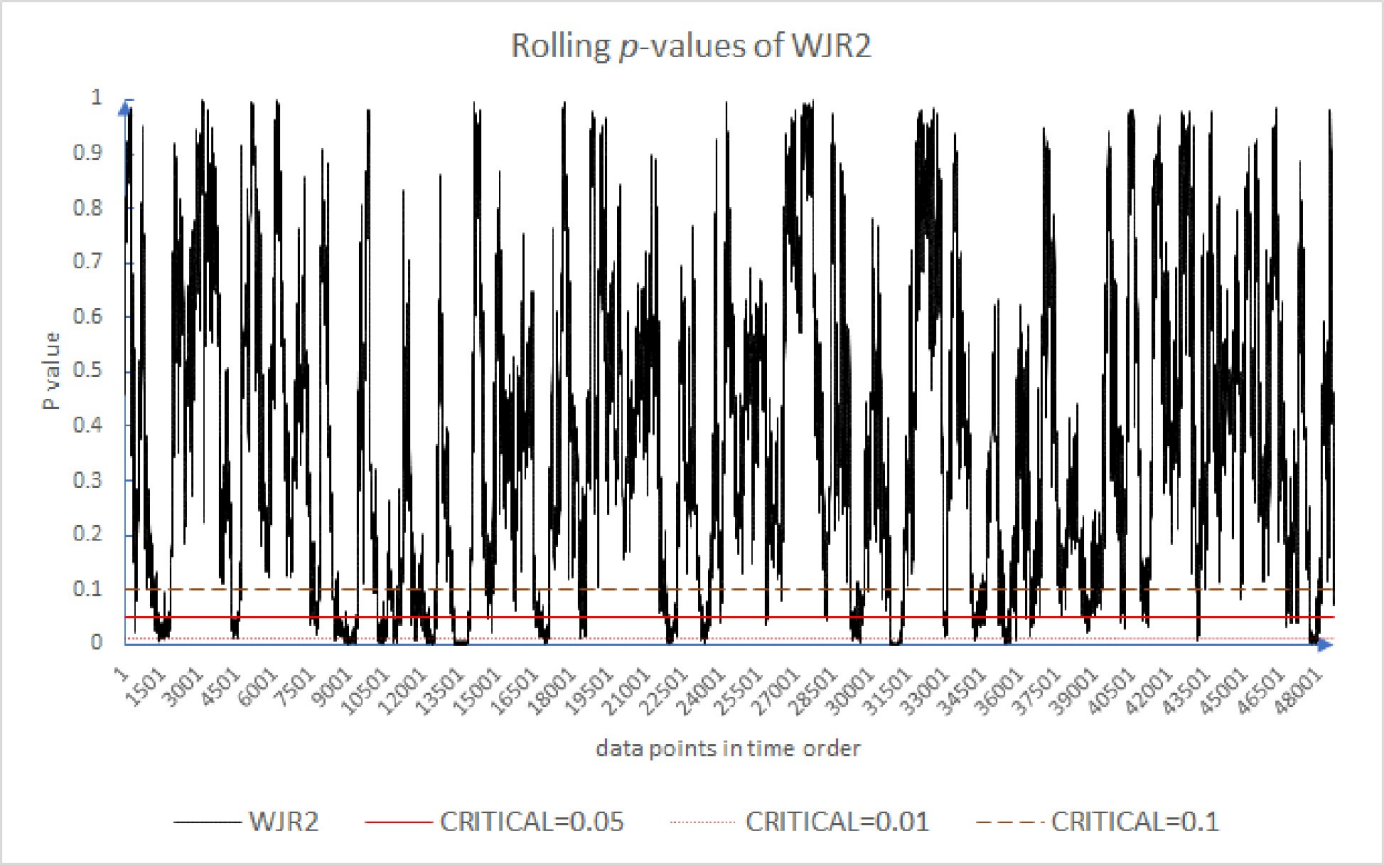
Moving *p*-value of WJR2 test for the first half year of 2012.

**Fig 4:**
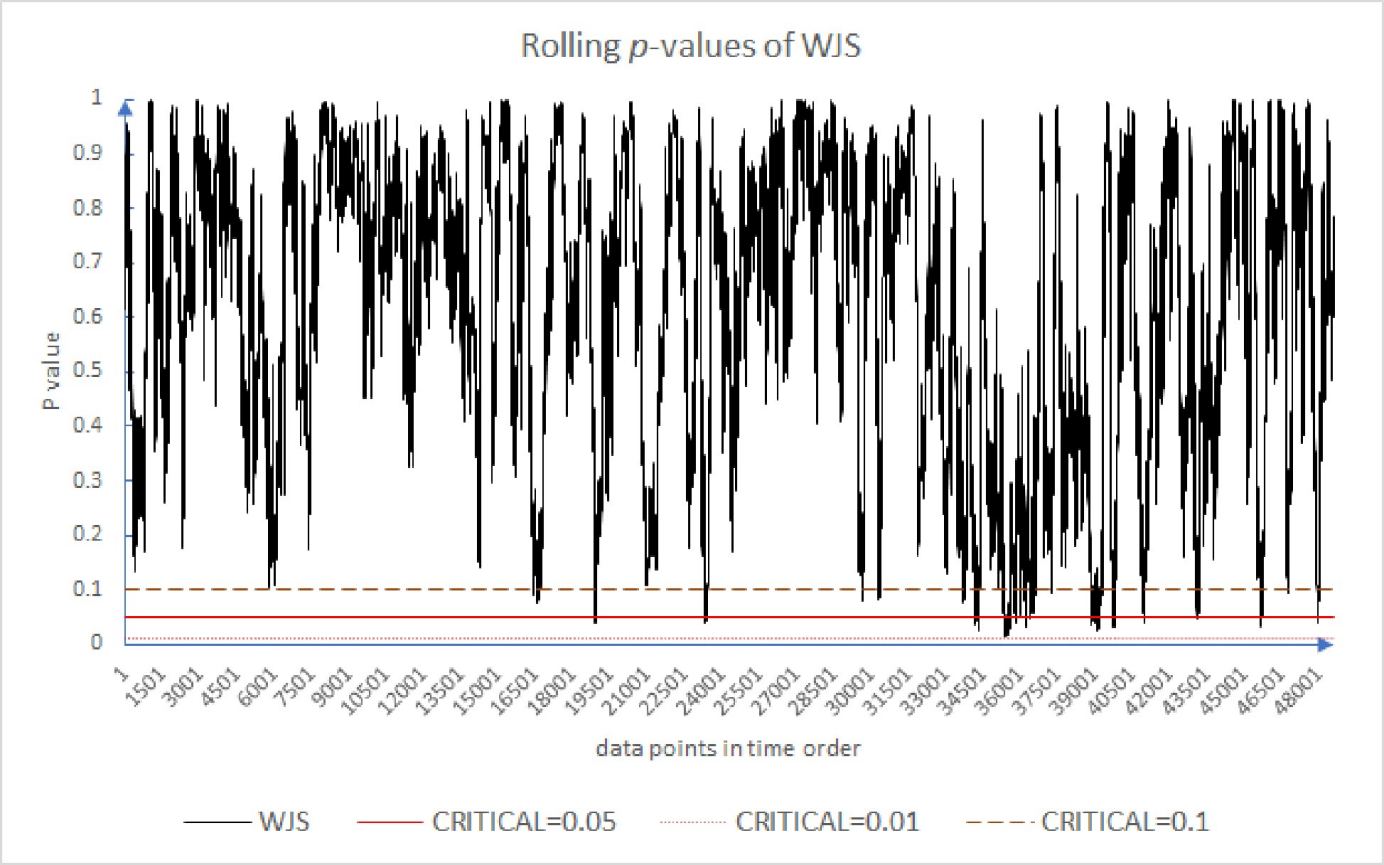
Moving *p*-value of WJS test.

Following the existing literature [5, 54, 56], we plot the results of the rolling *p*-values of the CDT, WJR1, WJR2, and WJS in Figs 1–4. For all these tests, we use the holding periods *k* = {2, 4, 8, 16}. We note that while the *p*-value may or may not exceed some *α* = 5% threshold, that is not our primary concern. Rather, our concern is the evolution of these *p*-values over time. Because of the large number of results, for brevity Figs 1 through 4 plot only the first half of 2012. As can be seen in Fig 1, the market appears to move from periods of high support for the EMH to low support and back again frequently. This is the inefficiency clustering discussed.

Similarly, Fig 2 presents the moving *p*-values of the WJR1 test for the first half of 2012. The frequency of *p*-values crossing the critical *α* = 5% is higher than with the CDT test in Fig 1. We can again see clusters of rejection or very low support for the efficiency hypothesis.

Fig 3 plots the results of the WJR2 test. We can see the similarity to Fig 2. Specifically, when the rolling *p*-values of WJR1 are low, those of WJR2 are low as well. We can see highly similar clustering. Though we know that these two tests use different distributions, this phenomenon is not unexpected since, through central limit theorem, the uniform distribution on which the WJR1 relies converges to the normal distribution used by WJR2.

With the WJS test, we get results that show greater efficiency. In Fig 4, the efficiency hypothesis is less frequently rejected at the *α* = 5% level. Though the rolling *p*-values evolve quickly, there are several points where the critical line is crossed.

Figs 1–4 provide evidence that over very short timeframes, the level of support for the EMH varies widely. All the results provide evidence that supports rejecting the EMH. Further, these results support the AMH idea of evolving efficiency. These results are robust since, over differing critical levels, 0.10 to 0.01, there are periods above and below the critical line. Even though Fig 4 has fewer inefficient periods, they are still evident. There appear to be periods of time where the market incorporates information relatively slowly, which are followed by periods where information is incorporated more quickly. Inefficient periods exist, but the market reverts to a higher level of efficiency.

Table 3 shows that the WJS test is least likely to reject the EMH as 99.31% of its rolling *p*-values exceed *α* = 5%, whereas the WJR test and CDT have 14.98% and 4.25% of their results reject the EMH respectively. The average of the results of these four tests suggests that 6.64% of the data points reject the EMH. Table 5 also compares our *one-minute* results with Urquhart and McGroarty’s *daily* results [5]. Similar to their results, our results are not consistent, something to be expected with noisy data. We can see that the percentage of the *p*-values less than *α* = 5% for the market at higher frequency is less than at lower frequency. This difference between the two is fairly consistent across all of our tests and is on average roughly 11.5%. The market appears to be more efficient at higher frequencies than at lower frequencies (To be clear, we are commenting here on the regularity in the time series pattern of p-values and the relative p-values at the various timeframes. And for comparison with the other study, the WJR test is averaged from WJR1 and WJR2. The average is taken for CDT, WJR, and WJS.).

**Table 3:** Proportion of significant results of overlapping windows.

Tests	% less than 0.05		Difference
	Our Results	Urquhart, *et al*. (2016)	
CDT	4.25%	13.70%	9.45%
WJR	14.97%	23.70%	8.73%
WJR1	18.12%	N/A	N/A
WJR2	13.76%	N/A	N/A
WJS	0.69%	17.04%	16.35%
**Average**	**6.64%**	**18.15%**	**11.51%**

### Results of HFT activity test using rolling *p*-values

In this section we examine the relationship between the level of support for the weak form of the EMH and the level of HFT activity. We regress the rolling *p*-values generated by these tests over the overlapping windows against our measure of HFT activity in Eq (5). We also use non-overlapping 30-minute windows and regress the rolling *p*-values against the high and low spikes in HFT activity.

First, for completeness we report the Pearson and Spearman’s rank correlations between the level of HFT activity as measured by (5), and the rolling *p*-values from the overlapping WJR, WJS, and CDT. In this analysis, we also control for market volatility by including the volatility index (VIX). The six-by-six correlation matrices are shown in Table 4, where all the results are significant at the 0.01 level. As we can see in Table 4, the rolling *p*-values all have positive correlations, though the correlations of WJS with the other three are quite low. And the correlation between WJR1 and WJR2 is very high as expected. The correlations between the *p*-values and HFT activity and the VIX are mixed.

**Table 4:** Correlation matrices of moving p-values.

**Panel A: Pearson Correlation Analysis**						
	**HFT**	**VIX**	**CDT**	**WJR1**	**WJR2**	**WJS**
**HFT**	1.0000					
**VIX**	-0.1346	1.0000				
**CDT**	-0.0401	-0.0942	1.0000			
**WJR1**	0.0881	-0.0736	0.2881	1.0000		
**WJR2**	0.0656	-0.0346	0.4230	0.8745	1.0000	
**WJS**	-0.1949	0.1036	0.0381	0.0681	0.0811	1.0000
**Panel B: Spearman Rank Correlation Analysis**						
	**HFT**	**VIX**	**CDT**	**WJR1**	**WJR2**	**WJS**
**HFT**	1.0000					
**VIX**	-0.1779	1.0000				
**CDT**	-0.0322	-0.0886	1.0000			
**WJR1**	0.1277	-0.0564	0.3334	1.0000		
**WJR2**	0.1115	-0.0439	0.4526	0.9030	1.0000	
**WJS**	-0.1763	0.0675	0.0413	0.0691	0.0584	1.0000

Second, we run multiple regressions for the rolling *p*-values against the level of HFT activity, while controlling for the VIX, trading volume, bid-ask spread, liquidity, and dummy variables for the opening half-hour and closing half-hour of trading. To reduce calculation load, we use only the first half of 2012, which is 48,641 data points. Calculating the rolling p-values for the whole sample took several weeks. For brevity, we report the results of the first half year. Since the calendar effect for traditional trading does not apply to HFT, this causes no bias. Table 5 reports the results of the four regressions and, since the correlations among the four tests are mixed, the mean of the *p*-values of the four tests is used for the fifth regression. This helps reconcile any discrepancy among the four tests.

**Table 5:** Regression analysis of rolling p-values.

Independent Variables	Dependent Variables				
	CDT	WJR1	WJR2	WJS	MEAN
**HFT**	-0.528***	0.726***	0.562***	-1.514***	-0.188***
	(-9.745)	(14.472)	(10.658)	(-31.346)	(-5.264)
**VIX**	-0.146***	-0.072***	-0.026***	0.068***	-0.044***
	(-22.959)	(-12.286)	(-4.225)	(12.054)	(-10.482)
**Trading Volume**	0.000	0.009***	0.005***	-0.026***	-0.003***
	(0.311)	(7.428)	(3.630)	(-21.743)	(-3.279)
**Spread**	94.534	274.079***	238.509***	-559.93***	11.798
	(1.507)	(4.718)	(3.901)	(-10.008)	(0.285)
**Liquidity**	0.213***	-0.326***	-0.328***	1.235***	0.199***
	(5.958)	(-9.830)	(-9.409)	(38.712)	(8.404)
**Open ½ Hour**	-0.010**	-0.011**	-0.017***	-0.004	-0.011***
	(-2.018)	(-2.299)	(-3.421)	(-0.973)	(-3.159)
**Close ½ Hour**	-0.003	-0.000	0.000	-0.018***	-0.005
	(-0.561)	(-0.080)	(0.031)	(-4.036)	(-1.591)
**Intercept**	1.159***	0.279***	0.318***	0.778***	0.634***
	(23.374)	(6.082)	(6.570)	(17.584)	(19.332)
***N***	48641	48641	48641	48641	48641
**Adj. *R*^2^**	0.012	0.015	0.007	0.085	0.005

Significance:

***0.01

**0.05

*0.1, with *t-*statistics in parenthesis

The lack of perfect correlation in across our tests of efficiency means that the regression results, although largely consistent among the measures, do show some conflicts. The role of HFT trading is always significant, but it is different for the WJR test than the VRT and WJS tests. This is not necessarily a defect in any one of the measures but more a pitfall of measuring statistics in a noisy system. This is the rationale for the fifth regression. We choose the mean of the *p-*values of the four tests because each test has its own strengths and weaknesses. The WJS test focusses only on one statistic but is very robust, while the CDT makes use of many features of the data but is more susceptible to higher moment deviations. A mean result generates a robust picture.

In Table 5, we focus on the negative relationship of -0.188 between the MEAN of the four measures of efficiency and the level of HFT activity. This value is significant in both the statistical sense and an economic sense. In Table 1 the range of HFT activity is 0.09 to 0.78. The negative sign on -0.188 implies that HFT activity tends to be higher when market efficiency is lower, and vice versa. This is consistent with the intuition that HFT traders look for opportunities to make money by providing efficiency services to the market. This is a significant contribution to the debate on the role of HFT.

Moreover, all the regressions except WJS show that efficiency is negatively related to VIX. For trading volume, looking at the MEAN regression, we see a negative coefficient of -0.003, suggesting that higher trading volume is associated with lower efficiency. This is similar to the VIX and suggests, similar to Miwa and Ueda that trading volume carries price information [58]. For Spread, there are no meaningful results. The Liquidity effect shown in the MEAN regression is positive, which suggests that when people are more willing to trade, efficiency is high. Finally, except WJS, none of the regressions find that the half hour prior to close plays a role, but they do agree that the first half hour after the open brings substantial information, which corroborates Blocher *et al*. [9]. Further, the opening half hour is negatively associated with efficiency.

Turning to the second measure of HFT effect on the market, we use the high and low spikes in HFT volume as the dependent variable against the rolling *p*-values for each non-overlapping 30-minute window for each test and the mean of the four tests. This tests the hypothesis that there are more positive (negative) outliers for HFT activity for periods when there is lower (higher) support for market efficiency. Table 6 reports the results of the five regressions using high spikes, where the dependent variable is the number of minutes out of 30 that have unusually high HFT activity. We also include control variables for the opening and closing half hours. In Table 6, regressions 1, 2, and 3 all give out significant negative relationships between the efficiency and high spike count. Though the result of WJS is insignificant, the relationship is the same. Further, of particular interest is the statistically significant negative relationship of -0.331 between the mean level of *p*-values of the four tests and the number of high spikes in HFT activity. These findings suggest that unusually high levels of HFT activity tend to occur when the market efficiency is low.

**Table 6:** Regressions of high HFT activity spikes.

	Dependent variable: High Spike Count				
Independent variables	Regression 1	Regression 2	Regression 3	Regression 4	Regression 5
**OPEN**	-0.215***	-0.200**	-0.204**	-0.209***	-0.207***
	(-2.697)	(-2.517)	(-2.566)	(-2.629)	(-2.604)
**CLOSE**	-0.086	-0.087	-0.088	-0.086	-0.089
	(-1.077)	(-1.094)	(-1.104)	(-1.085)	(-1.122)
**CDT**	-0.242***				
	(-3.395)				
**WJR1**		-0.228***			
		(-3.107)			
**WJR2**			-0.242***		
			(-3.282)		
**WJS**				-0.105	
				(-1.384)	
**MEAN**					-0.331***
					(-3.555)
**Intercept**	1.358***	1.339***	1.346***	1.281***	1.394***
	(30.434)	(31.655)	(31.659)	(28.815)	(26.794)
***N***	3237	3237	3237	3237	3237
**Adj. *R*^2^**	0.005	0.004	0.005	0.002	0.005

Significance:

***0.01,’

**0.05

*0.1, with *t-*statistics in parenthesis

Table 7 reports the results of the five regressions using low spikes. While most of the relationships are negative, they are not statistically significant. We conclude then that when there is little HFT activity, we cannot say much, if anything, about efficiency. In Table 6, the coefficients for opening half hour are all significant and negative while they are all conversely significant in Table 7. Though we know from other sources that the opening half hour contains the largest amount of information, and that HFT’s on average tend to be more active during this time [9], our results indicate they tend not to have upward spikes in activity during the open. They may, however, have a significant number of downward spikes, presumably to step out of the way of big price moves driven by information trades. Recalling from Table 5 that the opening half hour brings information and lower efficiency, the results from Table 7 seem to show that HFTs are aware of this and seek to avoid adverse selection by leaving the marketplace more often during this period.

**Table 7:** Regression low HFT activity spikes.

	Dependent variable: Low Spike Count				
Independent variables	Regression 1	Regression 2	Regression 3	Regression 4	Regression 5
**Open Hour**	0.338***	0.341***	0.340***	0.340***	0.339***
	(4.735)	(4.774)	(4.766)	(4.766)	(4.758)
**Close Hour**	0.054	0.053	0.053	0.056	0.053
	(0.751)	(0.749)	(0.747)	(0.780)	(0.749)
**CDT**	-0.054				
	(-0.836)				
**WJR1**		-0.042			
		(-0.644)			
**WJR2**			-0.045		
			(-0.679)		
**WJS**				0.059	
				(0.871)	
**MEAN**					-0.035
					(-0.423)
**Intercept**	0.866***	0.858***	0.859***	0.808***	0.855***
	(21.645)	(22.632)	(22.546)	(20.296)	(18.323)
***N***	3237	3237	3237	3237	3237
**Adj. *R*^2^**	0.006	0.006	0.006	0.006	0.006

Significance

***0.01

**0.05

*0.1, *t*-statistics in parenthesis

Across all our regression, while the *R*^2^ values are very low, all the relationships are very significant. However, explaining price movement is not our goal. Rather, we highlight the very specific effect of HFT activity on price efficiency. To summarize, though the regressions, especially in Table 5, do not reach a consensus regarding the direction of some variables to market efficiency, the significant results confirm the previous researches that return predictability is associated with evolving market conditions [1, 5].

## Conclusion

This paper examines the predictability of returns using one-minute time series data extracted from the limit order book of SPY. To our knowledge this is the first empirical study linking the activity of HFTs to market efficiency. Using well-known tests across the entire data set, we reject the EMH as a uniform hypothesis. By looking at overlapping sub-sample windows of data, we find that the level of support for the EMH varies widely over time, meaning that traders are adapting to information over time. This supports the argument that the AMH is more descriptive of returns than the EMH at this resolution, reinforcing the conclusion of Manahov *et al*. that the high frequency world is a Darwinian survival-of-the fittest ecosystem [7]. It is neither efficient nor inefficient, but requires the players to adapt to an evolving environment and compete to find and exploit opportunity. To understand the relationship between HFT and market efficiency over the short timeframes, we regress the measures of HFT activity against the *p*-values from the various tests. We find that while HFT activity explains only a very small amount of price movement, but there is a highly statistically significant negative relationship between them. Again, this seems to support the AMH. The results for other variables describing market conditions are significant, corroborating existing studies. Our results are consistent with the business model of most HFTs who pursue profit opportunities to remove inefficiencies when they arise, and act as passive market makers when the market is more efficient. Cooper *et al*. argue that HFTs invest in low latency technology only because they are able to generate sufficient return from trading [6]. In essence, this argument is that some market participants must be getting paid to keep the market at its level of efficiency or it would become less efficient. Another notable finding is that when information exists, there are more high spikes in HFT activity. This is consistent with the argument that HFT’s add-and-cancel activity is part of a rapid price discovery process when new information arrives as described in Cooper and Van Vliet [59].

The results in this paper open new questions for further research. For example, one question to investigate is the nature, or mechanism, of high frequency traders’ adaptiveness and how it allows them to survive. See also Manahov *et al*. [7]. Though it’s not realistic to expect traders to allow researchers to investigate their proprietary algorithms, it may be possible to ascertain their evolving logics over time given the ability to associate trades with specific traders. Then, a significant research program could test the contributions of those evolving algorithms to other evolving market factors, such as volatility, liquidity, and efficiency. For example, Hasbrouck anticipates that traders earn 0.1–0.4 cents of profit if they exclude the uncertainty of volatility in less than two seconds, which has not been empirically verified [60].

## Supporting information

S1 Fig Definitions of variables. (DOCX)

S1 Data (SAS7BDAT)
