# Correction: The adaptive market hypothesis and high frequency trading

**DOI:** 10.1371/journal.pone.0315633

**Published:** 2024-12-09

**Authors:** Ke Meng, Shouhao Li

There are errors in the Funding statement. The correct Funding statement is as follows: K.M. 2019T120111 China Postdoctoral Science Foundation https://jj.chinapostdoctor.org.cn/website/index.html. No role played.
