# Supplementary material for: The adaptive market hypothesis and high frequency trading
Source: PLoS One. 2021 Dec 17;16(12):e0260724. doi: 10.1371/journal.pone.0260724 (PMC8682897; doi:10.1371/journal.pone.0260724)
Supplement: S1 Fig — (DOCX) [file pone.0260724.s001.docx]

**Supporting Information**

| **Variables** | **Definition** |
| --- | --- |
| Return | The 1-minute bid-ask mid-point price log return, in percentage. |
| Trading Volume | The log of the trading volume in each interval |
| Liquidity | Liquidity level, the log of the level 1 volume over the log of minute mean volume (the minute level average traded volume of each day) |
| Intercept | The intercept of regression |
| VIX | The 1-minute log of VIXY level |
| Spread | The 1-minute bid-ask spread |
| WJS | The 1-minute moving p-values of Wright Joint Sign test |
| WJR1 | The 1-minute moving p-values of Wright Joint Rank test using *R*_1_ |
| WJR2 | The 1-minute moving p-values of Wright Joint Rank test using *R*_2_ |
| WJR | The mean of WJR1 and WJR2 |
| CDT | The 1-minute moving p-values of Chow-Denning test |

**S1 Fig. Definitions of Variables**
